# Gut *Bacteroides* species in health and disease

**DOI:** 10.1080/19490976.2020.1848158

**Published:** 2021-02-03

**Authors:** Hassan Zafar, Milton H. Saier

**Affiliations:** a Department of Molecular Biology, Division of Biological Sciences, University of California at San Diego, USA; b Department of Microbiology and Molecular Genetics, Faculty of Life Sciences, University of Okara,Okara, Punjab Pakistan

**Keywords:** Gut microbiome, Bacteroides, beneficial, pathogenic, carbohydrates, virulence factors

## Abstract

The functional diversity of the mammalian intestinal microbiome far exceeds that of the host organism, and microbial genes contribute substantially to the well-being of the host. However, beneficial gut organisms can also be pathogenic when present in the gut or other locations in the body. Among dominant beneficial bacteria are several species of *Bacteroides*, which metabolize polysaccharides and oligosaccharides, providing nutrition and vitamins to the host and other intestinal microbial residents. These topics and the specific organismal and molecular interactions that are known to be responsible for the beneficial and detrimental effects of *Bacteroides* species in humans comprise the focus of this review. The complexity of these interactions will be revealed.

## Introduction

The human gut is home to one of the most dense and diverse microbial communities known.^1^ The gene content of the gut microflora easily outnumbers that of the host by an astonishing 100-fold.^2^ The gut microbiome includes a plethora of biological entities including bacteria, viruses, fungi, archaea, and protozoa.^3,4^ The human colon is the main site of habitation for bacterial residents with an estimated concentration of 10^12^/ml.^4^

**Figure 1.**
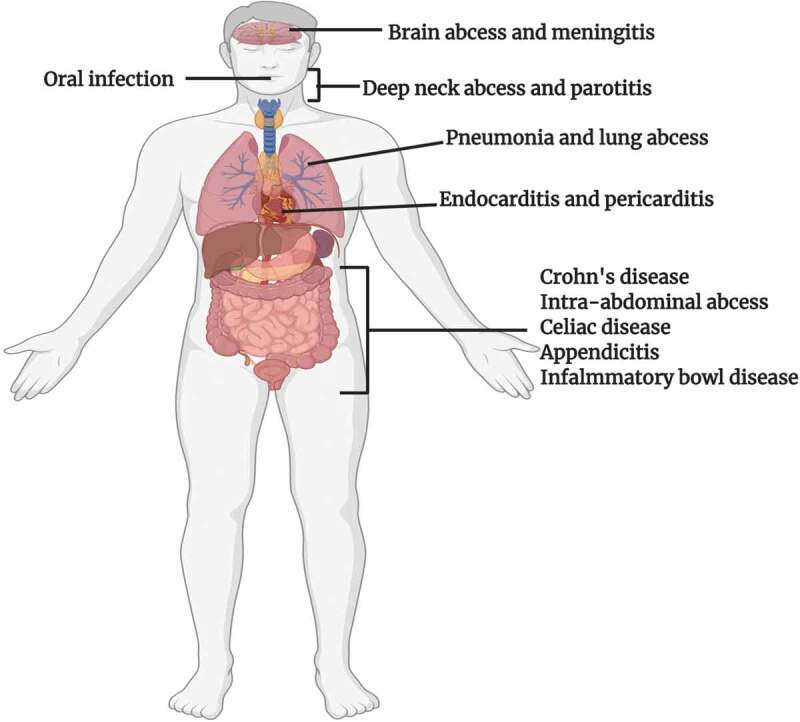
Sites of infection and diseases caused by *Bacteroides* spp

As the colon of the gastrointestinal tract (GIT) of mammals has the availability of diverse nutrient sources (derived from the host diet), this makes it a predilection site for numerous microbes.^5^ Members of the genus *Bacteroides* are potential colonizers of the colon and account for a major fraction of the gut bacteriome.^6^ These Gram-negative obligate anaerobes play multiple roles in the human gut bacteriome and are major players in sustaining the microbial food web of the gut.^7^ As proven commensals, mutualists, and beneficial organisms, they not only play the role of “Providers” for the host and other microbes residing close to them, but also assist the host by providing numerous health benefits. Nevertheless, some species of *Bacteroides* may play dual beneficial and pathogenic roles based on their locations in the host, often being beneficial in the gut but opportunistic pathogens in other body locations. Common sites of *Bacteroides* infections and possible disease conditions are illustrated in Figure 1. In this review we analyze the roles of *Bacteroides* species as beneficial organisms, gut competitors, and opportunistic pathogens. Also, recent relevant *Bacteroides* research findings will be evaluated.

*Bacteroides* spp. can cause infections in various parts of the human body. They have been isolated from numerous patients suffering from meningitis and brain abscesses. After entry into the blood stream during extraintestinal infections, these microbes may enter the CNS by penetration of the blood brain barrier via olfactory and trigeminal cranial nerves. They have also been associated with oral infections and abscesses in the neck. In 90% of the cases of lung abscesses, polymicrobial infection occurs, and *Bacteroides fragilis* has been the predominant anaerobe isolated. *Bacteroides vulgatus* and *Bacteroides fragilis* have been reported to be the two main isolates from patients suffering from Crohn’s disease, while the latter has been associated with intra-abdominal abscesses, appendicitis, and inflammatory bowel disease. Data adapted from Ref 7.

## 1. The human gut glycome and *bacteroides*

A major factor that shapes the composition and physiology of the gut bacteriome is the influx of glycans into the intestines via host diet and mucosal secretions.^8^ These glycans collectively form the human gut glycome, in which they are in constant interaction with each other and the microbial residents. The glycan landscape of the gut includes i) exogenous glycans derived from the host diet, ii) endogenous glycans expressed by the host cells, and iii) microbial glycans.^9^ Conjugated glycoproteins and glycolipids are examples of gut glycans, and these may be either O-linked (attached to serine or threonine residues) like host mucins, or N-linked (attached to asparagine), attached to cell surfaces as the glycocalyx, or unconjugated oligoglycans, which are often found in plants and fungi.^10^

In the human body mucus is present at the interface between many epithelial surfaces and their environments and is prevalent in the GIT.^11^ Interactions between the gut bacteriome and mucus are considered to be pivotal for the assembly and stability of the microbiota that reside in the gut. The main component of the mucus layers in the GIT are mucins which are O-glycosylated glycoproteins.^12^ The carbohydrate structures on these mucins are diverse due to which they can present microbes with a wide array of binding sites.^11^ Recent work has established that the mucin- rich mucus layer acts as an essential barrier between the luminal microbiota and the underlying immune cells.^13^

As gut commensals, *Bacteroides* spp. play multiple roles; they can provide protection from pathogens and supply nutrients to other microbial residents of the gut. Past research has revealed that mucin-type O-glycans are important contributors to their mutualistic roles and directly impact the interaction of *Bacteroides* spp with host tissues.^13^ The *Bacteroides thetaioatomicron* (Bth) VPI- 5482 strain has 88 polysaccharide utilization loci (PUL) at its disposal for the degradation of various kinds of glycans including diet derived and host glycans.^14^ The PUL of Bth enable it to effectively forage O-glycans during a shortage of glycans derived from plant polysaccharides.^15^ Generally, mucins are considered to be important players in the fitness and stability of *Bacteroides* spp. Binding of Bth to mucins and their subsequent degradation regulates the genetic repertoire that assists in the synthesis of the outer capsule. *B. fragilis* (Bfr), like Bth, has genetic machinery to degrade and utilize glycans, including mucin-type O-glycans, for capsular polysaccharide synthesis, which is collectively required for optimal colonization and maintenance in the gut.^16^ As Bth strains lack adhesive organelles, they utilize outer membrane glycan-binding proteins for attachment to food particles, mucus layers, and exfoliated epithelial cells.^17^ Bth has a flexible glycan-foraging ability, and it allows easy switching to host polysaccharides when dietary polysaccharides become scarce. This imparts an overall stability to the ecosystem of the gut bacteriome, during nutritional deficiencies.

Mucins primarily play protective and lubricative roles, but they also facilitate microbial tropism by presenting glycans to bacterial residents of the gut including *Bacteroides*.^18^ This, in turn, impacts the localization of these bacterial species and also gives them an extra nutritional source.^19^ As such, mucin glycans have been predicted to be key players in the selection and thriving of bacterial communities across the gut bacteriome. Consistent with this prediction, recent research using mouse models and humans indicates an association between changes in mucin glycosylation profiles and deviations of overall bacterial community ecology along with altered abundances of *Bacteroides* strains.^18^

## 2. Characteristics of polysaccharide utilization loci (PULs)

In the often nutrient-rich environment of the gut, one may assume that the microbes have easy access to desired nutrients, metabolizing them according to their physiological needs. However, in the colon, many of the desired nutrients, especially simple sugars, have already been absorbed and consumed in the small intestine. The remaining nutrients consist of long chain polysaccharides and oligosaccharides that are not readily absorbed by the epithelial cells of the colon, and resist digestion by host enzymes.^5^ For access to these lumenal carbohydrates, bacterial residents may require 1) extracellular polysaccharide hydrolases, 2) receptor proteins on the bacterial cell surfaces, 3) appropriate sugar transport systems, and 4) cytoplasmic carbohydrate degrading enzymes.^9,20,21^

**Figure 2.**
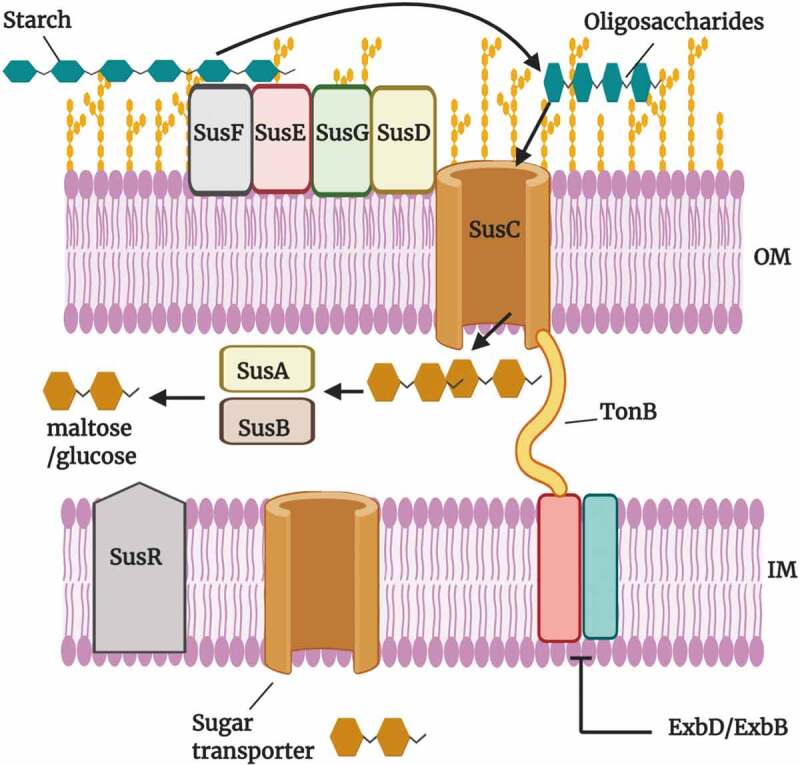
The starch utilization system (Sus) of *Bacteroides thetaiotaomicron.*

The polysaccharide utilization loci (PULs) of *Bacteroides* spp. may include secreted glycosidases, a complement of cell surface glycan-binding proteins, TonB-dependent outer membrane oligosaccharide receptor/transporters, uptake porters in the cytoplasmic membranes, and cytoplasmic carbohydrate-metabolic enzymes.^20^ Syntheses of these proteins are activated depending on the availability of carbohydrate sensors and transcriptional regulators. These sophisticated PULs provide the major protein machinery for carbohydrate acquisition and the initiation of metabolism in many *Bacteroides* species.^9^ These systems are pivotal to the colonization of nutritional niches and formation of gut-microbial ecosystems. One of the first identified PULs was the starch utilization system (Sus) of Bth.^21^ The Sus of Bth includes numerous cell-surface proteins (SusDEF), a TonB-dependent outer membrane transporter (SusC), and three enzymes (SusABG) as shown in Figure 2. The Sus proteins work in tandem for the capture and degradation of starch at the cell surface and for further digestion of the liberated malto-oligosaccharides in the periplasm. Many of the sequenced *Bacteroides* genomes possess PULs that show appreciable synteny with the Sus locus of Bth. However, there are some notable differences in the numbers of surface glycan-binding proteins like SusE and SusF. Also, the sequence similarities of these proteins differ as do the sizes of the predicted SusG hydrolase homologs.^21^

SusC is a TonB-dependent transporter that works in collaboration with the starch bindinglipoproteins SusD, SusE and SusF. These lipoproteins play roles in binding and immobilizing the extracellular starch polymers. Subsequently, SusG, an α-amylase, degrades the starch into smaller oligosaccharides which proceed to the periplasm via SusC. In the periplasm, SusA (α-amylase) and SusB (α-glucosidase) breakdown the oligosaccharides into maltose and glucose. These di- and mono-saccharides are transported into the cytoplasm via sugar transporting permeases. The sensor/regulator, SusR, (sensor domain in the periplasm; DNA binding domain in the cytoplasm) regulates the expression of the *susA-G* genes in response to maltose in the periplasm. The Sus system of Bth gives it an advantage in the competitive gut environment and also assists in the attachment to mucus glycans.

The use of the PUL machinery by *Bacteroides* spp. enables them to get involved in inter-species cross-feeding relationships with their microbial neighbors. It has been shown that Bth influences the dynamics of flavonoid degradation and butyrate production of *Eubacterium ramulus*.^22^ Flavonoids are phenolic compounds that are found in fruits and vegetables and arise as secondary metabolites in plants.^23^ Quercetin is one of the most well characterized flavonoids and is the most abundant one found in nature. It has numerous health benefits, being anti-viral, anti-inflammatory, antioxidant and anti-carcinogenic.^24^ Members of the gut microbiota, including *E. ramulus*, cleave the C-ring of quercetin during degradation and release 3,4-dihydroxyphenylacetate which has anti- proliferative activity in colon cancer cells.^25^ Bth seems to lack the metabolic machinery to degrade quercetin, while *E. ramulus* lacks the ability to degrade starch. Bth metabolizes starch (PUL mediated) and provides both maltose and glucose to *E. ramulus*. In the presence of these sugars, *E. ramulus* can degrade quercetin while fermenting glucose to butyrate.^22^ This PUL-mediated inter- species cross-feeding process is not only beneficial to gut residents for obtaining desired nutrients, it may also play beneficial roles for human health.

## 3. Outer membrane vesicles contribute to both health and disease

**Figure 3.**
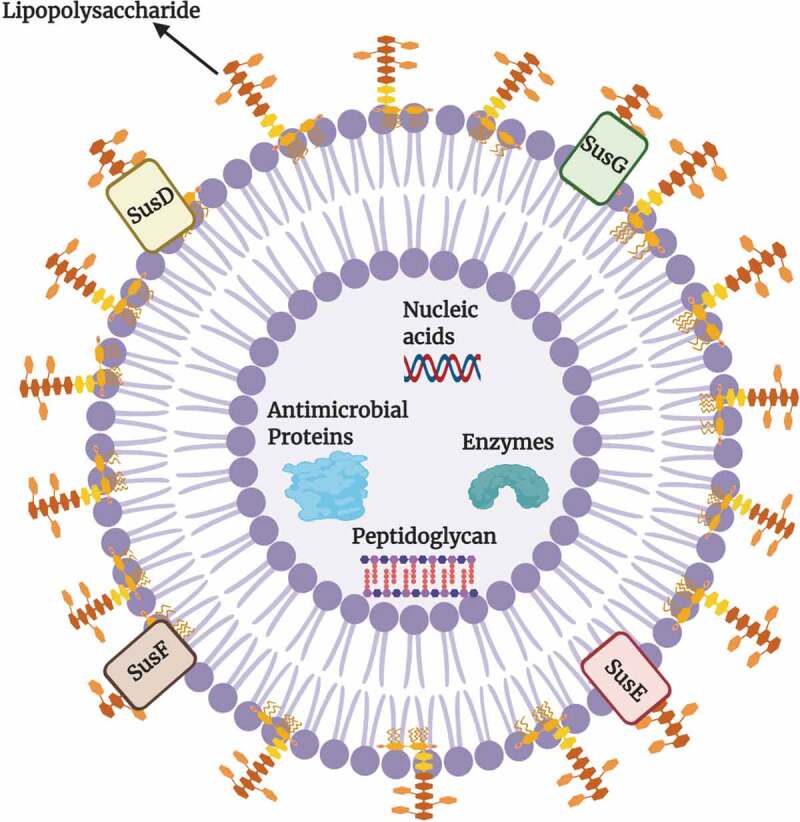
Structure of an Outer membrane vesicle (OMV) of *Bacteroides.*

Enteric Gram-negative pathogens are known to produce outer membrane vesicles (OMVs) containing mediators of virulence.^26^ These vesicles can be vehicles of pathogenicity as they can store and transport virulence factors over long distances.^27^ In addition to spreaders of virulence factors, research indicates that these OMVs can be recognized as key modes of communication between bacterial spp. and host tissues, contributing to a wide array of functions including i) nutrient uptake, ii) transfer of genetic material, iii) biofilm formation, and iv) protection from antimicrobials.^28^ *Bacteroides* have been shown to be major exporters of these OMVs with *B. fragilis* (Bfr) and Bth being two main players.^29,30^ The structure of a *Bacteroides* OMV is shown in Figure 3. The glycosidases, lipid hydrolases and proteases present in these vesicles help recipient bacterial species break down complex polysaccharides, proteins, and lipids to obtain monosaccharides and small oligosaccharides, amino acids and peptides, and fatty acids and other lipid breakdown products.^31^ As a result, the hydrolases of the OMVs play pivotal roles in the gut microbial ecosystem. By providing recipient bacteria (often called “cheaters”) with the required nutrients, the OMVs support the growth of other bacteria in the gut and contribute to overall gut homeostasis.^32^

The composition of the lipid bilayer of the OMV retains the same asymmetry as observed for the outer membrane of the parental bacterial cell. These vesicles can be carriers of a large repertoire of bacterial cargos such as i) nucleic acids, ii) fragments of peptidoglycan, iii) various enzymes such as glycoside hydrolases, iv) capsule components such as polysaccharide A (PSA), v) antimicrobial proteins such as Bacteriodales secreted antimicrobial proteins (BSAPs) that contain a membrane attack complex/perforin domain, and vi) Sus-like components. The OMVs of *Bacteroides* may contribute to both health and disease of the human host. These vesicles may transfer virulence factors to target cells residing in distant locations in the gut. The components of OMVs assist in the breakdown of complex polysaccharides, proteins, and lipids, thus supporting the growth of other bacteria and maintaining gut homeostasis.

The OMVs of Bth contain glycosyl hydrolases that help in the degradation of levan, a common non-structural carbohydrate in plants. The by-products of levan degradation include extracellular fructo-oligosaccharides that are important for the growth of other *Bacteroides* spp.^33–35^ Another example of OMV-associated intra-genus support involves *Bacteroides ovatus* and *Bacteroides vulgatus*. The glycosyl hydrolases in the OMVs of *B. ovatus* break down inulin, the products of which are utilized by *B. vulgatus*.^36^ Thus, the genus *Bacteroides* is an efficient public goods provider, and its services generally support other species in the microbial gut community.

## 4. Competition for shared nutrients in the gut

Gut microbes confront each other for available nutrients around them. *Bacteroides* spp. take part in exploitative competition and partially degrade polysaccharides for their own use instead of allowing other microbes to utilize them. For example, Bth only partially degrades the polysaccharide α-mannan and transports the products into its periplasm for further degradation into manno-oligosaccharides and mannose.^37^ As many other microbes do not encode the transporters and enzymes required for the use of these partially degraded polysaccharides, they can be used exclusively by the bacteria that do have them. This illustrates how Bth can use its metabolic repertoire to increase its competitive advantage over other gut residents.

### 4.1. Antimicrobial toxins are used in bacterial competition

*Bacteroides* spp. take part in interference competition by the secretion of antimicrobial toxins in a contact-independent manner. After secretion of diffusible toxins from the cell, *Bacteroides* spp. may utilize various transporters (such as ABC exporters) to actively secrete the toxins to the environment of their microbial targets.^38^ Several of the diffusible toxins produced by *Bacteroides* spp. have membrane attack complex/perforin (MACPF) domains. These domains are ubiquitous in eukaryotic cells and are involved in immunity and defense.^39^ The first recognized Bacteroidales secreted antimicrobial protein (BSAP-1) was found in approximately 44% of Bfr strains. As a lipoprotein, it is released as cargo within OMVs into the cell surroundings, and it kills target bacteria by pore-formation.^40^ BSAP-1 targets a β-barrel outer membrane protein of sensitive Bfr strains, thus exhibiting intraspecies killing.^41^ Bfr strains either possess the *bsap-1* gene encoding the toxin, or they lack the gene and are sensitive to it.^42^ Producers of BSAP-1 have a gene adjacent to the *bsap-1* gene that encodes an orthologue of the target outer membrane protein that serves as the receptor for BSAP-1 in the target bacterium.^43^ This orthologue is structurally similar to the target receptor but is sufficiently different, so as not to be targeted by the toxin, but to protect the producer from it. This renders the BSAP-1-producing strains resistant to a potential antimicrobial attack by its own toxin. In a mouse model of competitive colonization, it was shown that BSAP-1 producers have increased fitness in the presence of sensitive isogenic strains. Also, according to meta-genomic data, the co-residence of BSAP-1 producers and sensitive strains is a rare event in the mammalian gut, thus suggesting that BSAP-1 is a key tool for intra-species dominance *in vivo*.^41^ Two other MACPF toxins (BSAPs 2 and 3) are produced by *B. uniformis* and *B. dorei*/*B. vulgatus*, respectively.^43^ The mode of action of these two toxins may be similar to that of BSAP-1, but their receptor targets in the sensitive strains are different from that of BSAP-1, as they target lipopolysaccharides.^42^ Bfr also encodes a eukaryotic-like ubiquitin protein (BfUbb) that gives it a competitive advantage in the gut via intraspecies antagonism. Unlike BSAP 1–3, the mechanism of action is still unknown. Chaztidaki-lavanis *et al*. suggested a mechanism in which BfUbb is transported into target cells via a protein-specific uptake system that acts on an intracellular target rather than the outer membrane or lipopolysaccharide as is the case for BSAP 1–3.^44^ Recently, Shumaker *et al*. discovered another MACPF toxin called BSAP-4, which shows 42% similarity to BSAP-1. It targets outer membrane proteins with calycin-like domains that are exposed on the surfaces of target cells.^42^

### 4.2. Type vi secretion systems and contact dependent interbacterial antagonism

**Figure 4.**
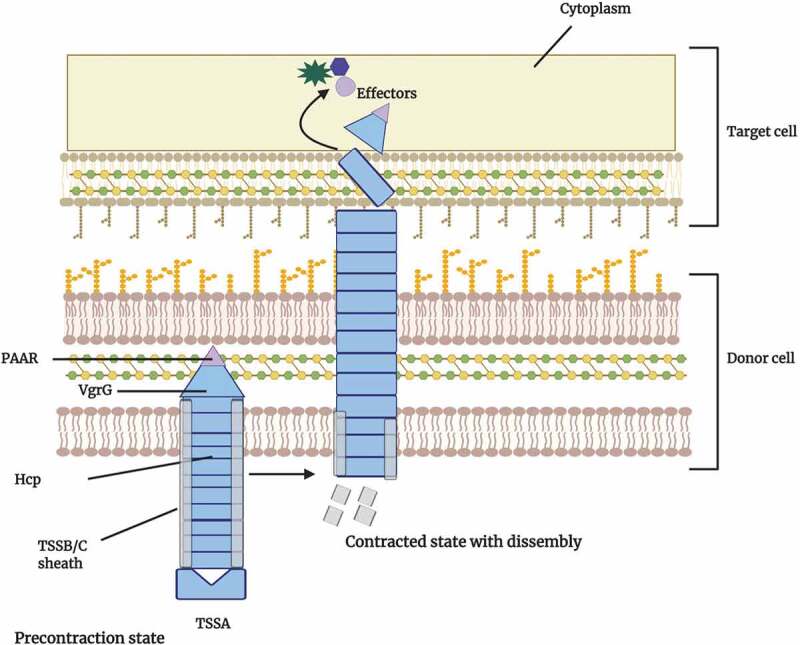
Model of the Bacteroidetes type VI secretion system

The generalized structures and topologies of Type VI secretion systems (T6SSs) are illustrated Figure 4 for*Bacteroides* spp. Upon direct contact with target cells, these multi-protein machines release antimicrobial toxins, effectors that mediate interbacterial antagonism.^45^ The T6SSs have structural and sequence similarity with the contractile tails of T4 bacteriophages, thus indicative of orthology between the systems.^46^ After the secretion of T6SS effectors by the bacterium, synthesis of specific immunity proteins confers resistance to potential attacks by sister cells. Each effector protein is accompanied by a cognate immunity protein, typically encoded by a neighboring gene.^47^

The type VI secretion system (T6SS) is a contractile nanomachine that injects antimicrobial proteins, termed effectors, into target cells. In the precontraction state of the system, the outer sheath of TSSB and TSSC multimers exists in a high energy state and covers the inner tube of Hcp multimers. When the outer sheath contracts to a lower energy state, the inner tube is forced out of the donor cell. At the end of the inner tube is the puncturing needle structure that contains a Valine Glycine Repeat G (VgrG) trimer and Proline-Alanine-Alanine-aRginine (PAAR) proteins that upon contact, are expelled into the target cell surface and cytoplasm. If the target cell lacks the cognate immunity proteins, death of the target organism may result. The T6SS assist *Bacteroides* spp. during interbacterial competition for nutrients with other commensals and pathogens and are predicted to be key players in intestinal homeostasis.

The T6SS loci of various *Bacteroides* spp. can segregate into three different genetic architectures, i) GA1, ii) GA2 and iii) GA3. The first two are present in numerous *Bacteroides* spp. while GA3 is exclusive to Bfr.^48^ The wide distribution of GA1 and GA2 is due to their presence on integrative conjugation elements which facilitate their spread to other species.^49^ Data from genomic and metagenomic analyses have revealed that the GA3 T6SS is found in 86% of Bfr strains.^50^ These GA3 T6SSs seem to confer upon Bfr a competitive advantage in the gut as they antagonize other *Bacteroides* spp. and *Parabacteroides* spp. However other Bfr strains with the same GA3 T6SS region and immunity genes survive.^51^ Analysis of infant bacteriomes showed that Bfr strains with GA3 structural genes are more prevalent (92%) compared to those in adults (74%).^52^ This suggests the GA3 T6SS enables Bfr strains to dominate competition with other bacterial spp. during early gut bacteriome development. Numerous studies have been conducted in attempts to understand the role of GA3 T6SS in the gut microbiota of mice. In germ free mice, Bfr strains with the GA3 T6SS outcompete Bth strains that lack it, thus suggesting that the system provides Bfr with a competitive advantage for colonization.^53^

The T6SS effectors of Proteobacteria are well characterized as are their targets in other bacterial cells including peptidoglycan, cell membranes and nucleic acids.^54^ These anti-bacterial effectors can be classified into families of nucleases, phospholipases, peptidoglycan hydrolases and NAD(P)^+^-dependent glycohydrolases.^54^ On the basis of homology, some putative effectors with similar functions have been predicted in *Bacteroides* spp., but many still remain unexplored with unknown functions and lacking recognizable domains. Due to a dependence on contact with the target cells, the target range of T6SS is limited to target cells within the immediate vicinity of the producers. T6SSs may prove to be important for *Bacteroides* spp. when competing locally for shared nutrients. This local action of T6SSs is in contrast to antimicrobial proteins which can exert effects on target cells at distant sites in the gut.

## 5. Prevalence of *bacteroides* species in children, adults and across different human populations

At birth, the gut is devoid of bacteria, but colonization starts shortly thereafter due to contact with the mother’s skin and environmental microbiota.^55^ Microbial colonization of the gut is also dependent on the type of delivery method used during birth, as *Bacteroides* spp. have been found to be prevalent in the gut of infants delivered vaginally.^56,57^ Also, the gut bacteriome of infants delivered vaginally has significant resemblance to their mothers. In the infant gut, the *Bacteroides* population is variably abundant, and infants fed with formula milk have a higher percentage of *Bacteroides* spp. as compared to the breast fed.^58^ Backheld *et al*. analyzed the development of the infant gut bacteriome during the first 12 months.^59^ By the age of 4 months, an increased production of amino acids and vitamins was observed. During the 10–12 months period the gut bacteriome of infants showed an increase in the expression of genes for the degradation of complex sugars. This was attributed to a higher abundance of Bth, known for its vast glycan degrading repertoire and ability to degrade human milk oligosaccharides.

Overall, microbial diversity of the gut reaches a fairly stable composition at the age of 3 years with the Bacteroidetes phylum as one of the three major phyla (Firmicutes and Actinobacteria being the other two).^60^ The introduction of solid food causes an increase in the bacterial load and diversity in the gut.^61^ This is due to higher total short-chain fatty acid levels, and a dominance of *Bacteroides* spp. that are adept degraders of complex glycans. Also, dietary habits such as high fiber and animal protein foods can cause an increase in the *Bacteroides* population.^62^

Various studies have been conducted to analyze the gut bacteriome in adults and children to get comparative insight into the respective microbiota. The most common techniques involved in these comparative studies have been 16S rRNA gene sequencing and shotgun metagenomic sequencing. Hollister *et al*. analyzed the gut microbiome of children of the age range of 7–12 years in Texas, USA by 16S rRNA sequencing and reported that members of the *Bacteroides* genus accounted for nearly 40% of the average healthy child gut bacteriome.^63^ Despite the comparative findings that both child and adult gut bacteriomes contain similar numbers of operational taxonomic units (OTUs), significant differences among the two groups were observed with respect to Shannon and Simpson diversity indices. Overall, *Bacteroides* was the common genus among children and adults with some species having a similar prevalence in the two groups. However, species that were more prevalent in the adult gut on the basis of 16S reads included *B. vulgatus* and *B. xylanisolvens*. Another study on the comparative gut composition of children (1–4 years) and adults in North Carolina, USA indicated that *Bacteroides* spp. were more prevalent in children.^64^ Zhong *et al*. examined the composition of the gut bacteriome of 281 school-going children (6–9 years) in the Netherlands.^65^ Variation among the prevalence of various *Bacteroides* spp. was observed. The most prevalent species on the basis of detected annotated genes were *B. ovatus* followed by Bfr, Bth and *B. xylanisolvens*. Interestingly, there was a larger prevalence of *Bacteroides* spp. in the gut of children.

The prevalence of *Bacteroides* spp. in the adult gut depends mainly on different factors such as diet, environment, and antibiotic use.^66^ However, important factors include dietary patterns, and the prevalence of species may vary in vegan, vegetarian and omnivorous diets. In a study based on the effects of dietary patterns on the gut bacteriome, Ferrocino *et al*. examined the fecal microbiota of 153 healthy volunteers (51 vegans, 51 vegetarians, and 51 omnivores) from four different locations in Italy.^67^ Bfr was present in lower numbers in both vegans and vegetarians but was highly prevalent in the omnivorous participants. Data from the V3 region of 16S rRNA gene sequences showed a prevalence of *B. salanitronis* and *B. coprocola* in the omnivorous group, while *B. vulgatus* was specific to the vegetarians and *B. salyersiae* was prevalent in vegans. The prevalence of *Bacteroides* spp. in general has been linked to animal-based diets; however, Bth is prevalent in vegans and vegetarians.^68–70^ Apparent discrepancies are common during categorization of certain bacterial spp. prevalence under a vegan/vegetarian diet vs an omnivorous diet. In this respect some *Bacteroides* spp. may play the role of outliers. This discrepancy in bacterial prevalence in different dietary patterns has been attributed to various causes, such as i) different methodologies for gut bacteriome profiling ii) variation in host genetics, iii) body mass index, and iv) consumption of red wine and aspartame (sugar substitute). ^69,70^

**Figure 5.**
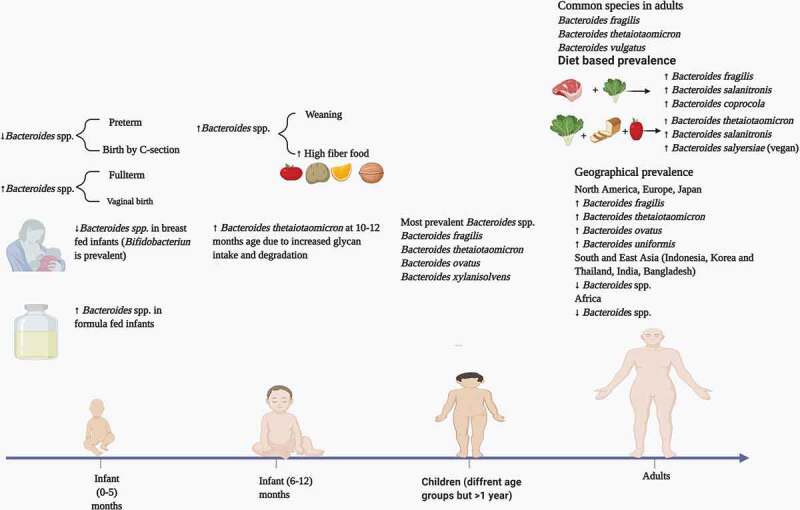
Prevalence of *Bacteroides* spp. in children, adults and across different geographical populations

The Gut bacteriome varies among individuals across different geographical locations around the globe. The overall structure of the gut bacteriome is influenced by different intrinsic and extrinsic factors such as, i) physiology and genetics of the host, ii) health and disease, iii) antibiotic use, and iv) diet.^71^ As dietary patterns vary across human populations in different geographical locations, so prevalence of *Bacteroides* spp. is also subject to variation. It has been demonstrated that *Bacteroides* species are prevalent in the guts of people living in Western countries (North America and Europe), as western diets are often high in fat and protein content.^72^ Prevalence of *Bacteroides* spp. in children, adults and across different geographical locations is summed up in Figure 5.

In new born babies the prevalence of *Bacteroides* spp. depends upon the mode of birth and type of milk consumed during the first five months. Between 6–12 months, *Bacteroides thetaiotaomicron* is the most prevalent specie; however, other species may increase in number during chilhood. In adults, dietary patterns are the most important factors, and the prevalence of *Bacteroides* spp. may vary in a western vs non-western diet. Data adapted from Ref 67, 68, 72, 73, 74 and 75.

Generally, in Asian countries, fat and protein consumption is minimal, and carbohydrates (in the form of rice and wheat) are consumed widely.^73^ However, due to diverse cultures and diet patterns, this cannot be considered as a trend as in many Asian populations, *Bacteroides* spp. have been observed to be present in significant numbers. For example, in a comparative study on the gut microbiota of populations from Japan and India, higher number of *Bacteroides* spp. were observed in Japanese participants who consumed a diet of animal origin in comparison to Indian adults who consumed a more plant-based diet.^74^ The most prevalent species in the Japanese adults were Bfr, Bth, *B. ovatus*, and *B. uniformis*. A study on the prevalence of *Bacteroides* spp. in Belgium adults showed an abundance of *B. uniformis, B. vulgatus* and *B. ovatus*, similar to the data concerning Japanese adult gut bacteriomes.^75^ Inhabitants of Indonesia, Korea and Thailand consume less meat and have a low prevalence of *Bacteroides* spp.^76^

## 6. *Bacteroides* and their pathogenic characteristics

*Bacteroides* spp. are generally ‘friendly’ commensals while residing in the gut, but they tend to become opportunistic pathogens when lodged elsewhere.^77^ For example, Bfr, a gut symbiont, has been found to be an opportunistic pathogen among the *Bacteroides* spp., as it is also the most common isolate from intra-abdominal abscesses.^78,79^ The translocation of *Bacteroides* spp. takes place through the intestinal mucosa into the normally sterile tissues, eventually producing different disease conditions.^8^ This translocation from the gut to extraintestinal locations in the body may be attributed to various factors: i) a compromised immune system, ii) gut barrier disruption (leaky gut), iii) surgical injuries, iv) excessive antibiotic use and v) aging.^80,81^ Also, dietary patterns may lead to a lack of competition by other gut commensals due to which an overgrowth of *Bacteroides s*pp. may occur. For example, an over-abundance of *Bacteroides caccae* results in the increased degradation of mucus, which helps reduce intestinal inflammation by decreasing bacterial interactions with intestinal epithelial cells in the intestine generally, and in the colonic mucus barrier specifically.^82^ Due to thinner layers of mucus, the intestinal barrier function becomes compromised, resulting in the expulsion of potential pathogens to extraintestinal locations. In addition, *Bacteroides* spp. may transfer virulence genes, thus equipping sister cells and neighbors with virulence factors that may assist in pathogenesis in extra-intestinal organs.^83^

Initially, during early infiltration in the extra-intestinal organs, aerobic bacteria dominate and cause tissue damage.^84^ Then, the redox potential of oxygenated tissues decreases, and anaerobes such as *Bacteroides* begin to thrive, leading to inflammation, diarrhea, and the formation of intra-abdominal abscesses.^8^ The dissemination of *Bacteroides* spp. outside of the gut lumen can thus lead to bacteremia and abscess formation in different body parts, sometimes even in the central nervous system.^85,^

### 6.1. Virulence factors of *Bacteroides*

*Bacteroides* spp. possess some of the most complex polysaccharide capsular systems among bacteria, consisting of at least eight different polysaccharides (PSA – PSH).^86^ A common function of the capsule is adherence to peritoneal surfaces. However, it also provides resistance to phagocytosis, thus playing an important role in bacterial fitness outside the colon.^87,88^ The lipopolysaccharides of *Bacteroides* spp. lack O-antigen and are approximately 1000 times less virulent than the lipopolysaccharide of *E. coli*.^89,90^ Like *Salmonella* spp., Bth chemically modifies the lipid A portion of the LPS, thereby increasing resistance to a subset of antimicrobial peptides.^91^

Bfr can be classified into two subtypes based on their pathogenic potential 1) non-enterotoxigenic strains that do not encode the Bfr toxin, and 2) enterotoxigenic Bfr strains that do have the *bft* genes that encode the toxin.^92^ Toxigenic strains of Bfr have been associated with different disease conditions of the human gut, including ulcerative colitis, toxin-mediated acute diarrhea, and bacteremia.^93,94^ The Bfr toxin (fragilysin) is one of the best-researched virulence factors among *Bacteroides* spp. It exists in three isoforms, BFT 1–3, with BFT-2 having the greatest potential to elicit tissue damage. Among isolates from humans, BFT-1 is the most common toxin variant, while the BFT-3 has a geographical propensity for Southeast Asia.^95,96^ As a heat-labile nickel ion-dependent metalloprotease, it shares substantial similarity with the tetanus and botulinum toxins. BFT is produced as a pre-protein and is cleaved by the fragipain cysteine protease to form the mature 20 kDa secreted toxin that is enterotoxic and cytotoxic in lamb ileal loop assays and HT29 cell lines.^97^

Proteases of the C10 family are pivotal virulence factors in a variety of bacterial species such as *Streptococcus pyogenes* and *Prevotella intermedia*.^98^ Thornton *et al*. reported homologues of the streptococcal virulence factor, SpeB, in Bfr with the encoding genes (*bfp* 1–4) located on mobile genetic elements, thus indicative of horizontal gene acquisition.^99^ A detailed understanding of the mechanism of action of these Bfr toxins will require future research, but due to structural similarity to SpeB, the proposed action includes cleavage of cytokines, immunoglobulins, extracytoplasmic matrix proteins and fibronectin.

Many Gram-positive and Gram-negative bacteria, produce hemolysins/cytolysins that are powerful virulence factors which lyse and kill host immune cells.^100–102^ These virulence factors not only contribute to the survival of the pathogens by providing access to nutrients but also weaken the immune system of the host.^103–105^ These hemolysins/cytolysins may be used by opportunistic pathogens to develop system infections in the host. Also, gut commensals may employ these toxins for advantage in the highly competitive gut environment. Robertson *et al*. identified ten hemolysin paralogs, HlyA to HlyI and HlyIII, encoded within the genome of Bfr.^104^ Further studies by Lobbo *et al*. showed that the expression of the hemolysins increases in an oxygen-rich environment and decreases during infection, mediated by the iron-dependent Fur transcriptional regulator. Bfr mutant strains (lacking genes *hlyA/B*) showed reduced fitness both *in vitro* and *in vivo*, suggesting that hemolysins may have roles in bacterial colonization of the gut.^83^ To date, clear evidence is lacking regarding the specific role of hemolysins in the pathogenesis of Bfr diseases. In our previous study on *Bacteroides* (Zafar and Saier, 2018), we observed disparate patterns of distribution of hemolysins among seven strains including Bfr and Bth.^105^ Only in the Bfr strain, did we observe a homologue of Hemolysin III, a powerful virulence determinant of *Bacillus cereus*.

The type 9 secretion system (T9SS) is a protein export pathway of the Fibrobacteres-Chlorobi-Bacteroidetes superphylum and has been associated with periodontal diseases in humans.^106^ The components of the T9SS complex are not similar in sequence to those of other well-studied bacterial secretion systems. Studies on these systems indicate their role in the secretion of virulence factors that damage human tissues and manipulate host immune responses.^107^ Other potential pathogenic functions include biofilm formation, adhesion and motility.^108,109^ So far, genomic studies have shown that components of T9SSs are present in a minority of the *Bacteroides* spp. including Bfr and Bth. However, the system is functional in the oral pathogen, *Bacteroides forsythia*, and the cargo proteins of its T9SS contribute to the evasion of host innate immunity.^110^ Future studies aimed at revealing the presence of these virulence-promoting systems in other *Bacteroides* spp. will be of considerable interest.

### 6.2. Oxidative stress responses as virulence factors and protective mechanisms

As gut residents, *Bacteroides* spp. are exposed to various oxygen concentrations. During extraintestinal infections, *Bacteroides* translocate to the more oxygenated (up to 7% O2) peritoneal cavity, where additional oxidative stress is exerted by the host immune response, including the recruitment of polymorphonucleocytes.^111,112^ The ability to survive oxidative stress is a key virulence factor,^113^ as pathogens must be able to withstand the conglomerate of oxidative host responses. This increased aerotolerance is achieved by the actions of various oxidoreductases including catalases, peroxidases and thioredoxins. In addition, the transcription factor OxyR is pivotal for the induction of numerous genes involved in oxidative stress response pathways.^114^

The Bfr genes *katA, ahpC* and *tpx* encode catalase, alkyl hydroperoxidase and thioredoxin peroxidase, respectively. These proteins assist in the oxidative stress response by detoxifying peroxides.^115^ Bacterioferritin co-migratory proteins are encoded within the genomes of numerous bacterial species. These proteins are members of the thiol-specific antioxidant protein family and play key roles in the prevention of free radical formation and resultant cellular oxidative damage.^116^ Studies with Bfr by Nicholson *et al*. suggested that the bacterioferritin co- migratory protein, encoded within the *recA* operon, may play a role in maintaining metabolic fitness and genomic integrity in response to oxidative stress.^117^ This may be accomplished by assisting in the reduction of hydroperoxides, thereby preventing lipid oxidation and DNA damage during oxidative stress.

### 6.3. Role of bacteroides species in oncogenesis

Recent research on the human gut microbiome has suggested that the microbiota play pivotal roles in the genesis of various types of cancer in humans.^118–120^ A dysbiotic gut is more prone to cancer, as pathogens can exert negative effects on the host’s physiology, metabolism, and immune system, thus promoting tumor growth. It has been shown that gut dysbiosis is apparently linked to the growth of both local and distal tumors in the host.^121^

Spermine oxidase is an FAD-dependent enzyme that oxidizes spermine and is generally important for the catabolism of polyamines in mammals.^122^ The oxidative products of spermine oxidase activity are spermidine, the reactive oxygen species, hydrogen peroxide, and the aldehyde, 3-aminopropanal, each with the potential to produce cellular damage and aggravate pathogenesis.^123^ With Bfr, activation of the host’s spermine oxidase can occur, which, in turn, generates hydrogen peroxide and other reactive oxygen species that contribute to DNA damage, increasing the prevalence of cancer.^124,125^

**Figure 6.**
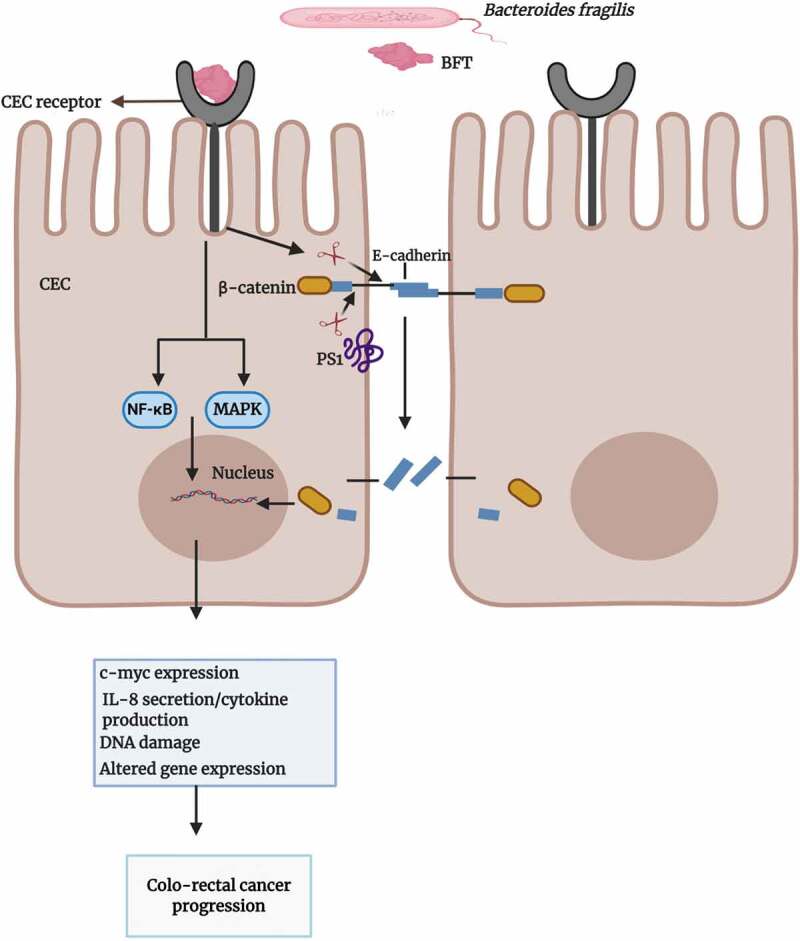
Overview of the potential role of BFT in colo-rectal cancer

Recent research on enterotoxigenic Bfr has shown it to be a major initiator and promoter of colo-rectal cancer in humans.^126^ The signaling pathways of colonic epithelial cells activated by Bfr is complicated with some mechanisms still being poorly understood. The zinc-dependent metalloprotease toxin of enterotoxigenic Bfr attaches to a colonic epithelial cell receptor and interacts with the host epithelial E-cadherin, a transmembrane protein essential for adhesion between colonic epithelial cells.^127,128^ Cleavage of E-cadherin results in the shedding of its 80- kDa extracellular ectodomain, followed by host cell presenilin-1/γ-secretase–mediated processing of the remaining intracellular fragment. This cleavage event leads to the disruption of intercellular junctions, activates the nuclear signaling protein, β-catenin, and induces the expression of the proto-oncogene, c-myc,^129^ as shown in Figure 6. The signaling mechanism may trigger cell proliferation and induce carcinogenic changes in the affected cells. BFT has been shown to activate nuclear factor kappa-light-chain-enhancer of activated B cells (NF-κB) and mitogen-activated protein kinase (MAPK) signaling pathways, leading to the release of interleukin-8 (IL-8) and tumor necrosis factor α (TNFα).^130,131^ IL-8 has been suggested to be a major player in tumor cell proliferation, tumor angiogenesis and growth.^132^

The interaction of the *Bacteroides fragilis* toxin (BFT) and the colonic epithelial cell (CEC) receptor results in E-cadherin cleavage which initiates a cascade of signaling events involving nuclear factor kappa-light-chain-enhancer of activated B cells (NF-kB), mitogen-activated protein kinase (MAPK) and β-catenin. Consequent cellular events of the signals include the proliferation of proinflammatory cytokines (such as IL-8) that promote proinflammatory microenvironments, expression of the proto-oncogene, c-myc, and damage to DNA. Overall, the cellular events triggered by BFT result in CEC proliferation, mucosal inflammation, and potential metastasis.

Around 5% of colorectal cancers occur in individuals who have an inherited mutation.^133^ Familial adenomatous polyposis is a hereditary condition caused by germline mutations in the adenomatous polyposis coli tumor suppressor gene.^134^ In this genetic background, numerous adenomatous polyps form in the epithelium of the colon, and malignant transformation may lead to colorectal cancer. Dejea *et al*. examined surgically resected tissue of patients with familial adenomatous polyposis and found that Bfr and *E. coli* (positive for the polyketide synthase (*pks*) island responsible for the synthesis of genotoxic colibactin) dominated the observed biofilms.^135^ Further experiments in mice showed that mono-colonization with either species resulted in few to no tumors. However, tumorigenesis was observed in the mice colonized with both species. *In vitro* trials using mucin monolayers indicated that degradation of mucin by Bfr enhanced colonization by *E. coli*. Thus, a shift in the niche of this species could facilitate the delivery of genotoxic colibactin to colonic epithelial cells, increasing the risk of mutations in genes such as that of adenomatous polyposis. This revealed another association of *Bacteroides* spp. with another gut resident, this time as a partner in crime for the initiation of colon tumorigenesis.

In another study, Toprak *et al*. detected the *bft* gene in stool samples of 38% of colo-rectal cancer patients, but in only 12% of samples taken from a healthy control group.^136^ Similar patterns of *bft* gene detection were reported by Haghi *et al*.^137^ Over the past decade, evidence obtained from next generation sequencing has suggested a relationship between the gut microbiome and breast cancer.^138^ This type of cancer is a global health concern as 1 in 8 women are expected to be affected in their lifetimes.^139^ Approximately 65% of breast cancers are diagnosed as hormone receptor positive (HR+), that in cancerous cells, have receptors for estrogen (ER+) and progesterone (PG+). A mouse model of hormone receptor-positive breast cancer suggested that prior gut dysbiosis in the mice led to a significant increase in tumor dissemination in the blood, lungs, and distal lymph nodes.^140^ This finding of tumor metastasis to distant sites supports the notion that the gut microbiome can be considered as an “endocrine gland”, at least when subjected to dysbiosis. Several bioactive metabolites secreted by gut microbial residents have been shown to affect immune cell functions and breast cancer cell growth in *in vitro* studies. Among these metabolites, short chain fatty acids, lithocholic acid, reactivated estrogen and cadverine can cause epithelial mesenchymal transitions and modulate mitochondrial metabolism in breast cancer.^141^

Relevant to the present review, *Bacteroides* spp. are efficient producers of these metabolites. A recent murine study of antibiotic-based gut dysbiosis showed that cephalexin (a cephalosporin-type antibiotic) accentuated the decrease in microbiome diversity that was induced by the tumor itself, and it induced tumor formation, indicating that antibiotic use and breast cancer incidence may be interlinked.^142^ This also supports the notion that a decrease of gut bacterial diversity correlates with cancer occurrence and metastasis. In the mouse gut, cephalexin decreased the number of butyrate producing bacteria including *Odoribacter* and *Anaeotruncus*, and increased the number of *Bacteroides* spp. As cephalexin is routinely used as a pre-surgery medication for breast cancer patients, future studies on the effects of other commonly used antibiotics on the gut microbiota will be of interest.

In a comparison of the gut microbiomes of healthy women and patients with invasive breast cancer, Cambell *et al*. reported increased levels of *Bacteroides* spp. in the cancer patients.^143^ In another study by Parida *et al*., mice infected with enterotoxigenic Bfr exhibited morphological changes in the mammary glands.^144^ Bfr toxin pretreated MCF7 cells exhibited increased tumor growth along with multifocal tumors in the mice models. MCF10A-KRas cells pretreated with Bfr toxin also showed increased tumor progression and multifocal tumors in mice. *In vivo* limiting dilution assays using breast tumors from Bfr toxin-pretreated MCF7 cells revealed a striking increase in tumor-initiating cells. Follow-up analyses of these tumors demonstrated increased migratory, invasive, and mammosphere-forming behavior, confirming that brief Bfr toxin exposure elicits long-term molecular changes in the cells.

### 6.4. Bacteroides and autoimmune disorders

The gut microbiota can be considered to be analogous to a fully functional organ of the body.^145^ Occasionally, the host relationship with the microbial residents of the gut has few defined boundaries. Thus, comes the issue of the commonality of epitopes of the gut residents and the human body. It is possible that under certain conditions, some gut microbes can give rise to autoimmune (immune reaction to self-epitopes) conditions.^146,147^

It has been hypothesized that molecular mimicry by proteins of bacteria and viruses in the human body may lead to autoimmune conditions.^148^ Recently, a shift in the symbiotic gut environment to a dysbiotic one has been linked to the occurrence of autoimmune conditions in humans.^149^ A case of molecular mimicry has been observed in the genome of Bfr, where the gene, *ubb*, encodes BfUbb. This protein has been found to be 63% identical to the human ubiquitin, Ubc52.^150^ Due to the similar molecular architecture of these two proteins, BfUbb may act as a mimic protein and generate antibodies that cross-react with self-epitopes. Ubiquitin plays several important physiological roles in the human body including regulation of the immune response and prevention of an autoimmune response to cellular debris by masking it.^151^ Doubtlessly, the generation of cross-reactive anti-ubiquitin antibodies could play a role in the onset of autoimmune conditions.^152^

Autoimmune inflammatory cardiomyopathy is a condition in which inflammation of the heart muscle is associated with impaired function of the myocardium.^153^ Different genetic and environmental factors may be responsible for the onset of this condition.^154^ However, a recent study by Gil-Cruz *et al*. has revealed that Bth may also play an important role in the occurrence of this condition.^155^ The authors found that Bth encodes a cross-reactive β-galactosidase mimic peptide, which can activate myosin-specific T-cells in the gut and can also induce a humoral response involving IgA and IgG in response to gut commensals. Briefly, they showed that inflammation occurring in the gut results in migration of the immune cells to the heart. This seems to trigger autoimmunity accompanied by cardiomyopathy.

## 7. *Bacteroides* as beneficial microbes for human health

*Bacteroides* spp. appear to be key players in the immunomodulation of the human immune system. Bfr expresses eight capsular polysaccharides, and the immunomodulatory effects of capsular polysaccharide A (PSA) have been the subject to extensive research.^156^ Documented beneficial effects of PSA in a nutshell include (i) stimulation, development, and homeostasis of the immune system,^157^ and (ii) prevention of bacterial and viral infections.^158,159^ PSA is packaged into OMVs and delivered to host cells.^160^

### 7.1. PSA of bacteroides fragilis, “an efficient immunomodulator”

Research on the effects of microbial peptides and carbohydrates on the immune system led to the concept that only peptides can induce adaptive T-cell responses. However, it is now established that zwitterionic-polysaccharides (carrying both negative and positive charges) such as PSA can induce CD4^+^ T cell–dependent immune responses.^161^ Toll like receptors (TLRs) comprise a family of transmembrane pattern recognition receptors that detect different but overlapping microbial components such as pathogen-associated molecular patterns (PAMPs), and they assist in eliminating these PAMPs host cells *via* signal transduction. TLR’s can reside on the cell surface (TLR’s 1,5,6 and 10) or localize to the endosome (TLR’s 3,7, 8, 9 and 11) although TLR2 and 4 are found in both compartments.^162,163^ Bfr uses the ability of its PSA to activate TLR2 signaling for localization in mucosal niches of the gut,^164^ while removal of TLR2 from CD4^+^ T cells results in an immune response against Bfr, thus limiting potential bacterial colonization.^165^ Overall, PSA influences CD4^+^ T-cell development, regulates the immune balance of T-helper cells (Th1/Th2), and activates immunomodulatory IL-10.^166^

Gut microbiota-virus interactions have been subject to extensive research in models of germ- free mice (born sterile and kept in a sterile environment) and antibiotic treated mice.^167^ The gut microbes may enhance, reduce, or have no effect on viral infections.^168^ These multifaceted roles in response to viral infections are possible by either direct modification of the virion or by immunomodulation of host responses.^169^ There has been recent interest in the role of *Bacteroides* spp. and their metabolites (specifically PSA) in equipping the immune system to combat viral infections. The role of PSA during Herpes simplex encephalitis (the most common type of fatal sporadic encephalitis in humans) was analyzed by Ramakrishna *et al*. in a murine model.^159^ This condition is caused by the herpes simplex virus 1 (HSV 1), and a review of past research on this type of pathogenesis indicates that during infection, immune pathology may lead to the uncontrolled dissemination of inflammatory neutrophils and monocytes into the brainstem.^170^ In the immunomodulatory analysis of PSA during viral infection, pre-treatment of mice with PSA was followed by infection with HSV1 and delayed treatment with Acyclovir, the antiviral drug of choice. The PSA-treated mice exhibited high survival rates as compared to controls (pre-treatment with PBS), and decreased levels of brainstem inflammation were also observed. A comparison with other mice that lacked B-cells and IL-10 showed that IL-10 was the main anti-inflammatory factor secreted by CD4^+^ and CD8^+^ T-cells. These T-cells seem to be induced by the binding of the PSA to B-cells. This suggests that PSA of Bfr may provide robust protective anti-inflammatory responses during viral infections.

### 7.2. Other bacteroides-mediated benefits

Metabolites secreted by different *Bacteroides* spp. assist in maintaining stability of the immune system. These species are primary producers of short-chain fatty acids in the human gut, mostly in the form of acetate and propionate. These are important for the maintenance of intestinal homeostasis.^171^ Both acetate and propionate are potent anti-inflammatory mediators as they inhibit the release of pro-inflammatory cytokines from neutrophils and macrophages.^172^ Cruz-Bravo *et al*. described an anti-cancerous role of propionate which induced apoptosis in human colon carcinoma cells.^173^ Also, butyrate increases expression of tight-junction proteins in the gut to reduce potential gut hyperpermeability. This, in turn, decreases inflammation and endotoxemia that are associated with leaky gut.^174^ In the human gut, *Bacteroides* spp. are the principal synthesizers of Vitamin K, which is mainly produced by members of the human gut bacteriome.^175^ It may prevent or treat osteoporosis by increasing the bone mineral density.^176^

## 8. Conclusions

*Bacteroides* can be regarded as the quarterback of a human gut football team. They try to pass the best available nutrients and beneficial metabolites to their teammates. Like a quarterback played out of position, a *Bacteroides* specie will not be able to perform its advantageous roles, and in turn will start eliciting adverse effects on the performance of its most important teammate, the human host. Thus, in the proper body location and under appropriate environmental conditions, it will be a good friend, but in an inappropriate location, it may become a foe. It is amazing how these bacterial spp. are equipped with such sophisticated metabolic machinery that enables them to perform various roles (commensals, beneficial microbes, and opportunistic pathogens), reflective of an extended co-evolutionary process. In this regard, it is important to note how these bacteria can influence cancerous cell growth either positively or negatively. Some strains of Bfr may be promoters of various types of cancers, by inducing different physiological changes in the host that may result in DNA damage. Also, enterotoxigenic Bfr strains have proven to be carriers of cancer-promoting toxins in humans. In addition, Bfr may enhance the colonization of certain enteric pathogens in individuals with hereditary conditions leading to the formation of certain cancer types.^135^ Another mechanism that may lead to cancer formation in the colon involves biofilms produced by certain *Bacteroides* spp. As this microbial structure may be formed in the ascending colon and contains certain metabolites (such as polyamines), it may increase the concentrations of reactive oxygens species.^177^

*Bacteroides* spp. are also considered to be key players in cancer immunotherapy and prevention. For example, *B. xylanisolvens* DSM 23964 has been shown to promote the maturation of natural antibodies against various cancers in humans.^178^ Another commensal strain, *B. ovatus* ELH-B2, has shown promise as a vaccine candidate for the development of a Thomsen-Friedenreich antigen (TFα)-specific anti-tumor vaccine.^179^ In a nutshell the properties of cancer prevention and immunotherapy among these organisms could be related to the type of strains used and their functional capacities.

Further research on the role of these species as, “potential anticancer probiotics” could lead to improvements in existing cancer therapies. Future research will help to reveal physiological and metabolic details about the less well understood *Bacteroides* strains and their interactions. The role of polysaccharide A of Bfr, for example, in immunomodulation during viral infection is a fascinating but underdeveloped subject. Investigations focusing on its robust, protective, anti-inflammatory roles during various viral infections will certainly be a topic of increasing interest.
